# Supplementary material for: High-Efficiency Achromatic Metalens Topologically Optimized in the Visible
Source: Nanomaterials (Basel). 2023 Feb 27;13(5):890. doi: 10.3390/nano13050890 (PMC10005494; doi:10.3390/nano13050890)
Supplement: Supplementary file 1 [file nanomaterials-13-00890-s001.zip › nanomaterials-2208070-supplementary.pdf]

nanomaterials

MDPI

Supplementary Material

High-Efficiency Achromatic Metalens Topologically Optimized in the Visible

Lijuan Zhang [1,2], Chengmiao Wang [1,*], Yupei Wei [1,2], Yu Lin [1], Yeming Han [1] and Yongbo Deng [1,*]

1 Changchun Institute of Optics, Fine Mechanics and Physics (CIOMP), Chinese Academy of Sciences, Changchun 130033, China
2 University of Chinese Academy of Sciences, Beijing 100039, China
* Correspondence: wang985504950@163.com (C.W.); dengyb@ciomp.ac.cn (Y.D.)

S1 Forward Problem

The electric field is calculated by solving wave problem:

$$\begin{aligned} \nabla\times\left[\mu_r^{-1}\nabla\times\left(\mathbf{E}_s+\mathbf{E}_i\right)\right]-k_0^2\varepsilon_r\left(\mathbf{E}_s+\mathbf{E}_i\right)=0,\ \text{in }\Omega \\ \nabla\cdot\left[\varepsilon_r\left(\mathbf{E}_s+\mathbf{E}_i\right)\right]=0,\ \text{in }\Omega \end{aligned} \quad (1)$$

Where the electric field split into two parts, the background field $\mathbf{E}_i$ and scattered field $\mathbf{E}_s$. The boundary conditions also usually include electric conditions:

$$n\times\left(\mathbf{E}_s+\mathbf{E}_i\right)=0,\ \text{on }\partial\Omega \quad (2)$$

The divergence-free condition in Equation **Error! Reference source not found.** must consider the gradient of the relative permittivity, which always arises in the topology optimization procedure. The permittivity gradient could result in the inapplicability of numerical solution method. To circumvent this problem, the computational domain Ω is assumed to be piecewise homogeneous. Because of this assumption, the relative permittivity is a constant in every piecewise domain:

$$\varepsilon_r\left(\Omega_n\right)=\text{const},\ n=1,2,\dots N \quad (3)$$

where Ω_n is a homogeneous piece of the computational domain, satisfying

$$\Omega=\bigcup_{n=1}^{N}\Omega_n;\ \Omega_p\bigcap\Omega_q=\varnothing,\ \text{with } p\neq q,\ \text{and } p,q=1,2,\dots N. \quad (4)$$

where N is the number of homogeneous pieces included in the computational domain. Based on the assumed piecewise homogeneity, the divergence-free condition in Equation **Error! Reference source not found.** can be transformed into:

$$\nabla\cdot E_s=0,\ \text{in }\Omega\ . \quad (5)$$

S2 Topology Optimization Problem and Adjoint Analysis

The filtered design variable is calculated by using the Helmholtz filter equation:

$$\begin{aligned} -r^2\nabla\cdot\nabla\gamma_f+\gamma_f=\gamma \\ \boldsymbol{n}\cdot\nabla\gamma_f=0 \end{aligned} \quad (6)$$

where γ is the material density, γ_f is the filtered material density. Based on the assumed piecewise homogeneity, the filtered material density γ_f is transferred into a piecewise version:

$$\gamma^e = \sum_{n=1}^{N} \gamma_n^e(\Omega) \tag{7}$$

where

$$\gamma_n^e(\Omega) = \begin{cases} \frac{1}{V_{\Omega_n}} \int_{\Omega_n} \gamma_f d\Omega, \ \forall \mathrm{x} \in \Omega_n \\ 0, \ \forall \mathrm{x} \in \Omega_n \end{cases} \tag{8}$$

where $V_{\Omega n}$ is the volume of Ω_n. To make the optimized structure boundary clear, the filtered design variable is projected:

$$\gamma_p^e(\gamma^e) = \frac{\tanh(\beta\zeta) + \tanh\left(\beta\left(\gamma^e - \zeta\right)\right)}{\tanh(\beta\zeta) + \tanh\left(\beta(1-\zeta)\right)} \tag{9}$$

where γ_p^e is the projected material density; ζ is the threshold, which is generally 0.5; β is the projection parameter. The initial value of the projection parameter β is set to be 1 and it is doubled after every fixed number of iterations until the preset maximal value 1024 is reached (11 cycles). A linear interpolation formula for air and dielectric materials in the design domain:

$$\varepsilon_r\left(\gamma_p\right) = \varepsilon_{ra} + \gamma_p^e\left(\varepsilon_{rm} - \varepsilon_{ra}\right), \text{ in } \Omega_d \tag{10}$$

where ε_{ra} and ε_{rm} are the relative permittivities of air and dielectric materials, respectively. The variational problem for the topology optimization of the electric field can be formulated as:

$$\begin{aligned} &\min: J\left(\mathbf{E}_s, \nabla\times\mathbf{E}_s, \gamma_p^e; \gamma\right) \\ &\text{s.t.:} \begin{cases} \nabla\times\left[\mu_r^{-1}\nabla\times\left(\mathbf{E}_s + \mathbf{E}_i\right) - k_0^2\varepsilon_r\left(\mathbf{E}_s + \mathbf{E}_i\right)\right] = 0, \text{ in}\Omega \\ \nabla\cdot\mathbf{E}_s = 0, \text{ in}\Omega \\ -r^2\nabla\cdot\nabla\gamma_f + \gamma_f = \gamma, \text{ in}\Omega \\ 0 \le \gamma \le 1 \end{cases} \end{aligned} \tag{11}$$

where Ω is the piecewise homogeneous computational domain; *J* is a formulated cost functional

$$J\left(\mathbf{E}_s, \nabla\times\mathbf{E}_s, \gamma_p^e; \gamma\right) = \int_\Omega A\left(\mathbf{E}_s, \nabla\times\mathbf{E}_s, \gamma_p^e; \gamma\right) d\Omega \tag{12}$$

where A is the integral functional of the field variables.

According to the Kurash-Kuhn-Tucker condition of the PDE constrained optimization problem, the adjoint equations can be obtained as: find $\mathbf{E}_{sa} \in \mathcal{V}_E$ and γ_{fa} in $\mathcal{H}^1(\Omega)$, satisfying

$$\int_\Omega \frac{\partial A}{\partial \mathbf{E}_s}\cdot\phi + \frac{\partial A}{\partial\nabla\times\mathbf{E}_s}\cdot(\nabla\times\phi) + \mu_r^{-1}\left(\nabla\times\overline{\mathbf{E}}_{sa}\right)\cdot(\nabla\times\phi) - k_0^2\varepsilon_r\overline{\mathbf{E}}_{sa}\cdot\phi d\Omega \tag{13}$$

and

$$\int_\Omega r^2\nabla\overline{\gamma}_{fa}\cdot\nabla\phi + \overline{\gamma}_{fa}\phi + A_{\gamma^e}\phi - S_{\gamma^e}\phi d\Omega = 0, \ \forall\phi\in\mathcal{H}^1(\Omega) \tag{14}$$

where $\mathbf{E}_{sa}$ and γ_{fa} are the adjoint variables of $\mathbf{E}_s$ and γ_f, respectively; $A_{\gamma^e}(\Omega)$ is defined to be

$$A_{\gamma^e} = \sum_{n=1}^{N} A_{\gamma_n^e}(\Omega_n) \tag{15}$$

where

$$A_{\gamma_n^e}(\Omega_n) = \begin{cases} \frac{1}{V_{\Omega_n}} \int_{\Omega_n} \frac{\partial A}{\partial \gamma_p^e} \frac{\partial \gamma_p^e}{\partial \gamma^e} d\Omega, \ \forall \mathrm{x} \in \Omega_n \\ 0, \ \forall \mathrm{x} \in \Omega \setminus \Omega_n \end{cases} \tag{16}$$

and $S_{\gamma^e}(\Omega)$ is defined to be

$$S_{\gamma^e} = \sum_{n=1}^{N} S_{\gamma_n^e}(\Omega_n) \tag{17}$$

and

$$S_{\gamma_n^e}(\Omega_n) = \begin{cases} \frac{1}{V_{\Omega_n}} \int_{\Omega_n} k_0^2 \frac{\partial \varepsilon_r}{\partial \gamma_p^e} \frac{\partial \gamma_p^e}{\partial \gamma^e} (\mathbf{E}_s + \mathbf{E}_i) \cdot \bar{\mathbf{E}}_{sa} d\Omega, \ \forall \mathrm{x} \in \Omega_n \\ 0, \ \forall \mathrm{x} \in \Omega \backslash \Omega_n \end{cases} \tag{18}$$

The adjoint derivative of the cost functional can be derived as

$$\delta J = \int_{\Omega} \left(\frac{\partial A}{\partial \gamma} - \bar{\gamma}_{fa} \right) \delta \gamma d\Omega \tag{19}$$

The topology optimization method for three-dimensional electric field is implemented by a gradient-based iterative procedure, where the gradient information is derived by sensitivity analysis. The iterative procedure includes the following steps: (i) solve the wave equations with the current design variable; (ii) solve the adjoint equations based on the solution of the wave equations; (iii) compute the adjoint derivative of the design objective; and (iv) update design variables using the method of moving asymptotes (MMA). The iterative procedure stops, when the change of the objective values in five consecutive iterations satisfying

$$\frac{1}{5} \sum_{n=1}^{4} \frac{|J_{k-1} - J_{k-i-1}|}{|J_k|} \le \varepsilon, \beta \ge 1024 \tag{20}$$

in the kth iteration, where J_k is the objective value in the kth iteration; ε is the tolerance chosen to be 1×10^{-6}.

S3 Design Process

The problem of the topology optimization of the electric field:

$$\begin{aligned} &\min F = \sum_m \left| \mathbf{E}_{srm} - \alpha_m e^{i\varphi_{tm}} \right|^2, \text{ in } \Omega_d; \\ &\text{s.t. } \nabla \times \left[\mu_r^{-1} \nabla \times (\mathbf{E}_s + \mathbf{E}_i) \right] - k_0^2 \varepsilon_r (\mathbf{E}_s + \mathbf{E}_i) = 0, \text{ in } \Omega \ ; \\ &\qquad \nabla \cdot \mathbf{E}_s = 0, \text{ in } \Omega \end{aligned} \tag{21}$$

where $k_0 = 2\pi/\lambda$ is the free space wave number; ε_r and μ_r are the relative permittivity and relative permeability of the nano-post, respectively; $\mathbf{E}_{sm}$ and $\mathbf{E}_{im}$ are the scattered and background field, respectively; m=1, 2, 3 … represents the case for the m-th wavelength; $\mathbf{E}_{srm}$ is the electric field of the cross-polarized light; $\alpha_m \cdot \exp(i \cdot \varphi_{tm})$ is the target electric field of the cross-polarized light; α_m and φ_{tm} represent the amplitude and target phase, respectively. So the adjoint derivative of the cost functional can be derived as

$$\delta J = \int_{\Omega} -\gamma_{fa} \delta \gamma d\Omega \tag{22}$$

The phase is linearly related with the frequency of the nano-post in the range from 531 to 780 nm, so two wavelengths are selected to input into the topology optimization model of nano-post in order to reduce the calculation amount. The wavelengths are 600 nm and 650nm, respectively. Where 600 nm is set as the design wavelength. With only two wavelengths, there may be a problem that the optimized group delay moves away from target group delay caused by 2π resetting. However, the group delays of the optimized nano-posts are in the range from 3fs to 7.5fs. Beyond this range, the phase can not be linearly related with the frequency of the nano-post. And in this range, the 2π resetting problem can be avoided. So the target group delays should be in this range.

Twenty values are set from 0 to 2π at intervals of 0.1π as the basic phase φ_0 at the designed wavelength. Based on the basic phase and target group delay, the target phases of two wavelengths are calculated and substituted into the topology optimization model. The target amplitudes of two wavelengths are both 0.9. In the process that the amplitude values are changed to make the objective function approaches 0, there are many optimized nano-posts with high polarization conversion efficiencies, so these nano-posts are placed in the library. The group delay and average polarization conversion efficiency of the nano-post in the library as shown in Table 1. And More details about the structures of the nano-posts can refer to https://pan.baidu.com/s/1t6bJh-aOjtBIbuik6Ikysg.

Table S1. Group delay and average polarization conversion efficiency of the nano-post in the library.

Number	Group Delay(fs)	Average Polarization Conversion Efficiency	Number	Group Delay(fs)	Average Polarization Conversion Efficiency
1	7.17	0.66	2	7.34	0.68
3	7.02	0.64	4	6.90	0.59
5	3.83	0.33	6	2.93	0.27
7	3.63	0.31	8	3.82	0.43
9	4.6	0.57	10	4.50	0.57
11	3.79	0.33	12	7.11	0.63
13	6.73	0.53	14	6.50	0.70
15	6.52	0.64	16	6.96	0.50
17	7.19	0.59	18	6.95	0.58
19	6.81	0.51	20	6.89	0.37
21	3.41	0.21	22	3.07	0.26
23	3.21	0.19	24	6.59	0.54
25	6.26	0.28	26	6.43	0.28
27	6.82	0.32	28	6.58	0.37
29	6.46	0.68	30	7.14	0.55
31	7.05	0.41	32	7.10	0.33
33	7.05	0.56	34	5.30	0.52
35	3.23	0.24	36	3.66	0.27
37	3.10	0.16	38	5.41	0.45
39	4.69	0.49	40	4.41	0.35
41	3.85	0.41	42	7.06	0.50
43	6.96	0.56	44	6.94	0.35
45	7.05	0.17	46	5.93	0.50
47	6.96	0.46	48	7.12	0.39
49	7.15	0.39	50	7.03	0.46

51	7.20	0.66	52	7.20	0.68
53	6.84	0.55	54	6.98	0.63
55	7.28	0.63	56	3.12	0.54
57	6.31	0.18	58	7.44	0.26
59	7.50	0.22	60	7.07	0.36
61	7.24	0.59	62	7.11	0.61
63	7.21	0.68	64	7.17	0.66
65	7.21	0.25	66	7.17	0.57
67	6.72	0.51	68	6.98	0.58
69	6.62	0.56	70	7.30	0.66
71	7.18	0.70	72	6.19	0.63
**Number**	**Group Delay(fs)**	**Polarization Conversion Efficiency**	**Number**	**Group Delay(fs)**	**Polarization Conversion Efficiency**
73	5.81	0.66	74	5.72	0.65
75	5.35	0.60	76	4.83	0.22
77	5.91	0.54	78	5.97	0.26
79	5.74	0.28	80	5.85	0.65
81	5.55	0.63	82	5.48	0.61
83	5.34	0.47	84	4.70	0.48
85	4.33	0.44	86	4.15	0.67
87	4.96	0.48	88	4.81	0.44
89	6.43	0.61	90	7.09	0.66
91	7.06	0.64	92	7.07	0.66
93	6.85	0.25	94	6.25	0.56
95	7.04	0.26	96	6.99	0.51
97	7.23	0.25	98	6.66	0.51
99	6.48	0.64	100	7.15	0.64
101	7.10	0.67	102	7.26	0.66
103	5.04	0.46	104	6.75	0.44
105	6.03	0.24	106	6.03	0.24
107	6.75	0.58	108	4.90	0.19
109	4.00	0.20	110	2.89	0.55
111	4.12	0.22	112	4.54	0.60
113	6.28	0.59	114	7.14	0.64
115	7.40	0.55	116	5.64	0.58
117	5.87	0.47	118	4.49	0.30
119	5.46	0.18	120	4.75	0.30
121	5.76	0.49	122	7.06	0.59
